# Low-Dose Paclitaxel Ameliorates Pulmonary Fibrosis by Suppressing TGF-β1/Smad3 Pathway via miR-140 Upregulation

**DOI:** 10.1371/journal.pone.0070725

**Published:** 2013-08-15

**Authors:** Congjie Wang, Xiaodong Song, Youjie Li, Fang Han, Shuyan Gao, Xiaozhi Wang, Shuyang Xie, Changjun Lv

**Affiliations:** 1 Department of Clinical Medicine, Binzhou Medical University, Yantai, China; 2 Medicine Research Center, Binzhou Medical University, Yantai, China; 3 Key Laboratory of Tumor Molecular Biology in Binzhou Medical University, Department of Biochemistry and Molecular Biology, Binzhou Medical University, Yantai, China; 4 Department of Respiratory Medicine, Affiliated Hospital of Binzhou Medical University, Binzhou, China; Helmholtz Zentrum München/Ludwig-Maximilians-University Munich, Germany

## Abstract

Abnormal TGF-β1/Smad3 activation plays an important role in the pathogenesis of pulmonary fibrosis, which can be prevented by paclitaxel (PTX). This study aimed to investigate an antifibrotic effect of the low-dose PTX (10 to 50 nM in vitro, and 0.6 mg/kg in vivo). PTX treatment resulted in phenotype reversion of epithelial-mesenchymal transition (EMT) in alveolar epithelial cells (AECs) with increase of miR-140. PTX resulted in an amelioration of bleomycin (BLM)-induced pulmonary fibrosis in rats with reduction of the wet lung weight to body weight ratios and the collagen deposition. Our results further demonstrated that PTX inhibited the effect of TGF-β1 on regulating the expression of Smad3 and phosphorylated Smad3 (p-Smad3), and restored the levels of E-cadherin, vimentin and α-SMA. Moreover, lower miR-140 levels were found in idiopathic pulmonary fibrosis (IPF) patients, TGF-β1-treated AECs and BLM-instilled rat lungs. Through decreasing Smad3/p-Smad3 expression and upregulating miR-140, PTX treatment could significantly reverse the EMT of AECs and prevent pulmonary fibrosis of rats. The action of PTX to ameliorate TGF-β1-induced EMT was promoted by miR-140, which increased E-cadherin levels and reduced the expression of vimentin, Smad3 and p-Smad3. Collectively, our results demonstrate that low-dose PTX prevents pulmonary fibrosis by suppressing the TGF-β1/Smad3 pathway via upregulating miR-140.

## Introduction

Pulmonary fibrosis, a progressive and usually devastating fibrotic lung disease, is characterized by phenotypic dys-transition in the alveolar epithelial cells (AECs) with extracellular matrix collagen deposition. Numerous microRNAs (miRNAs) and their targeted genes were identified as regulatory factors of EMT, a process of emerging importance in IPF [1]. Transforming growth factor-β1 (TGF-β1) is recognized as a “master switch” to induce fibrosis, as well as EMT and myofibroblast generation. The direct targets in TGF-β1 pathway, Smads (Smad2, and especially Smad3), were critical mediators in fibrogenesis and EMT [2], [3]. The signaling activity of Smad3 is modulated via phosphorylation and cytosol-nucleus translocation. The lung phenotype in pulmonary fibrosis is regulated by aberrant recapitulation of the TGF-β1/Smad3 pathway. Inhibition of Smad3 or phosphorylated Smad3 (p-Smad3) resists TGF-β1-induced EMT and fibrosis. By binding Smad3 mRNA 3′-untranslated region (3′-UTR), miR-140 can downregulate Smad3 expression directly [4], [5]. Recent studies confirm that the TGF-β1 pathway is suppressed by miR-140 through targeting Smad3 in the C3H10T1/2 and 3T3 cell lines [4], [5]. However, the role of miR-140-related TGF-β1/Smad3 pathway in pulmonary fibrogenesis remains unclear.

Pulmonary fibrosis management is highly debatable, and no effectively curative treatment has been developed so far. PTX is used to block dynamic cytoskeletal processes to stabilize cellular microtubules (MTs), which are involved in a wide range of cellular biological processes, including division, migration, maintenance, and intracellular trafficking of organelles [6]. PTX activity has been clinically exploited for anti-tumor therapy. Recently, researchers have shown that low-dose PTX inhibits collagen-induced arthritis, hepatic fibrosis, and fibrosis associated with systemic sclerosis [6]–[8]. PTX can also significantly relieve tubulointerstitial fibrosis in a rat unilateral ureteral obstruction model and a remnant kidney model [9], [10]. Moreover, TGF-β1/Smad3 signals play a central role in IPF by interacting with the microtubular network [11]. Thus, we speculate that PTX has a protective role in relieving pulmonary fibrosis. Then, we treated TGF-β1-stimulated AECs and bleomycin (BLM)-induced pulmonary fibreosis rats with PTX to verify the antifibrotic effect of PTX on pulmonary fibrosis and to clarify the underlying mechanisms involved in the TGF-β1/Smad3 pathway.

## Results

### Effect of PTX on inhibiting EMT in AECs

**Figure 1:**
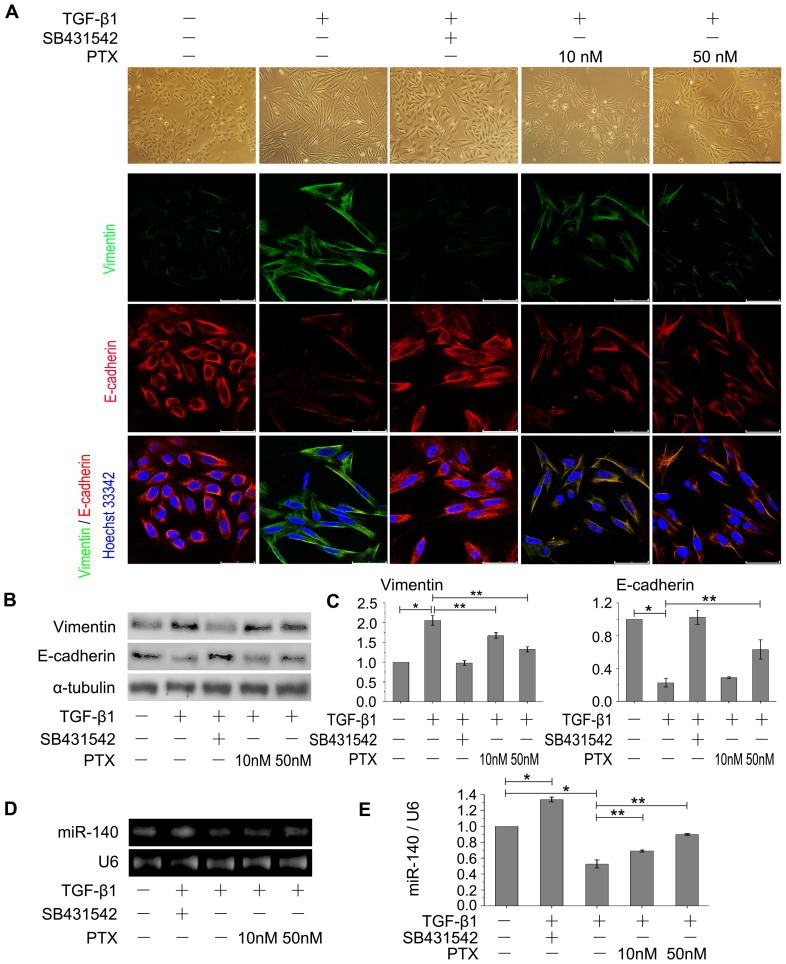
PTX ameliorates EMT and upregulates miR-140 in A549 cells. A: Cell morphological changes and fluorescence staining. The TGF-β1-treated A549 cells exhibited morphological changes of EMT, converting from their epithelial phenotype into fibroblastic phenotype, with diminished E-cadherin and increased vimentin expression, while PTX reversed the EMT phenotype and its marker proteins, especially in 50 nM PTX-treated cells. Red, CY3-labeled E-cadherin; Green, FITC-labeled vimentin. Scale bars: 150 µm (upper), 50 µm (lower). B: Western blot analysis. α-tubulin was used as loading controls, n = 3 replicates. C: Quantitative RT-PCR analysis. GAPDH was used as a control gene, n = 3 replicates. *P<0.05 versus untreated control cells. **P<0.05 versus TGF-β1-treated cells. In Fig. 1 B, C, PTX treatment resulted in a reversal of TGF-β1 action by upregulating E-cadherin and decreasing vimentin levels. D: MiR-140 expression is determined by RT-PCR. n = 3 replicates. E: Quantitative RT-PCR analysis of miR-140 expression. n = 3 replicates. The miR-140 expression was significantly reduced in TGF-β1 treated A549 cells, while was increased dramatically after PTX treatment (Fig. 1 D, E). *P<0.05 versus untreated control group. **P<0.05 versus TGF-β1-treated cells.

Low doses of PTX (10 and 50 nM) were used in this study according to previous reports [6]–[10], which showed that low-dose PTX could inhibit multi-organs fibrosis. Because TGF-β1-triggered EMT is a key issue in the pathogenesis of pulmonary fibrosis [12]–[14], we investigated whether PTX could ameliorate EMT through TGF-β1 pathway. Loss of E-cadherin, a central target of transcriptional regulators, is a universal feature of EMT [15]–[17]. Reversion of the invasive mesenchymal phenotype can be observed if E-cadherin is constitutively produced [17], [18]. Vimentin, a general fibroblast marker for the meshchymal cells derived from epithelium, is usually used to reflect the degree of EMT [16], [19]. Downregulation of E-cadherin and upregulation of vimentin are directly related to EMT [17]. Thus, the expression of E-cadherin and vimentin was investigated after TGF-β1 treatment, and we found that alveolar epithelium-derived A549 cells exhibited an EMT in response to 5 ng/mL TGF-β1, converting from their epithelial phenotype to fibroblastic phenotype, with reduction of E-cadherin and overexpression of vimentin (Figure 1 A–C). After TGF-β1 treatment, A549 cells displayed stellate and elongated fibroblast-like morphologies, with low cell-cell contact and high primary expression of the mesenchymal marker (vimentin). When treated with TGF-β1 signal pathway blocker SB431542, A549 cells exhibited a cobblestone-like epithelial morphology, with normal cell-cell adhesion and primary expression of the epithelial marker (E-cadherin), similar to the morphologies of control cultures. These results proved the important role of TGF-β1 pathway in EMT.

To study the effect of PTX on EMT changes, A549 were treated by TGF-β1 with/without PTX. Our results showed that TGF-β1 induced a hyperplastic cell phenotype of EMT and PTX could resist the action of TGF-β1 in A549 cells. PTX treated A549 cells exhibited mostly intact and nearly normal epithelial morphologies (Figure 1 A), with primary expression of E-cadherin and notably vimentin suppression (Figure 1 A–C). To further test the effect of PTX on resisting EMT in another epithelial cell line, RLE-6TN cells were added TGF-β1 with/without PTX. Our results also demonstrated that PTX could prevent EMT induced by TGF-β1 in RLE-6TN cells (Figure S1). The above study supports that PTX reverses EMT phenotype and suppresses the activity of TGF-β1.

### Effect of PTX on ameliorating pulmonary fibrosis in rats

**Figure 2:**
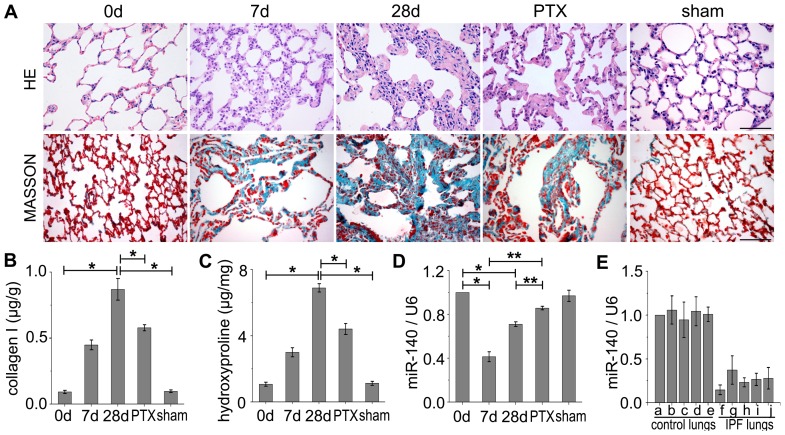
PTX ameliorates pulmonary fibrosis andu pregulates miR-140 in fibrotic lungs. A: Upper, Haematoxylin and eosin (HE). The lungs initiated alveolar disruption on 7 d, and more severe on 28 d, while pulmonary fibrosis was ameliorated by PTX (0.6 mg/kg) treatment. Lower, Masson's trichrome for collagen and elastin evaluation. The lungs underwent a slight fibrotic invasion on 7 d, and more aggressive on 28 d, which was alleviated by PTX. Scale bars: 150 µm. B: Type-I collagen ELISA analysis. The type-I collagen level showed basally low in lung tissues of rats instilled with saline (sham control rats), similar to those in the untreated lungs (0 d). The collagen I levels in BLM-treated lung tissues on 28 d were increased seven folds than those in untreated or sham control lung tissues. Collagen I levels were reduced obviously in PTX-treated lungs compared to BLM-treated 28 d lung tissues. *P<0.05 versus 28 d. C: Hydroxyproline analysis. BLM-treated lungs had much higher levels of hydroxyproline, but PTX treatment significantly decreased hydroxyproline levels compared to BLM-treated 28 d lungs. *P<0.05 versus 28 d. D: Quantitative RT-PCR analysis of miR-140 expression. U6 was used as a control. n = 3 replicates. The miR-140 level in BLM-instilled rat lungs reached its nadir on 7 d, approximately two folds lower than that in untreated or sham control lung tissues, while it was dramatically upregulated in PTX-treated lung tissues. *P<0.05 versus 0 d. **P<0.05 versus PTX. E: miR-140 expression in human pulmonary fibrotic lungs. a–e: healthy control lungs, f–j: human pulmonary fibrosis lungs. The miR-140 levels are obvious lower in humanulmonary fibrotic lung tissues compared with those in healthy control lungs.

BLM, a mixture of glycopeptides derived from Streptomyces verticillus, is known to produce pulmonary fibrosis in humans as well as in experimental animals [20]–[24]. It is generally believed that BLM itself causes direct injury to epithelial or endothelial cells in the lung [25]. BLM-elicited fibrotic lung injury has been extensively used to investigate the mechanisms involved in the pathogenesis of pulmonary fibrosis [21], [22], [26], [27]. To further characterize the function of PTX on relieving pulmonary fibrosis, we used BLM to produce pulmonary fibrotic model of rats. Low-dose PTX (0.6 mg/kg) was performed according to Brahn's study [6]. First, we found that PTX had no side effect on the changes of lung phenotype in rats, which showed that the lung phenotype of the saline+PTX-treated rats was almost the same as that in the saline-treated rats (Figure S2). Then, we investigated the roles of PTX in resisting the BLM-induced pulmonary fibrosis. The wet lung weight is an indicator of lung inflammation [28]. We found that the BLM-treated rats lost body weights, increased lung-to-body weight ratios and the pulmonary inflammation and fibrosis scores, which could be ameliorated by PTX (Table 1). Moreover, the BLM-induced pulmonary fibrotic lungs underwent a severe epithelial degeneration, alveolar disruption, and initiated fibrotic invasion on day 7, and more prominent distortion of the alveolar architecture as well as more thickening of interalveolar septa were found on day 28. No significantly pathologic changes were found in the only saline-treated rats (sham control) (Figure 2 A). However, we found an attenuated interalveolar collagen deposition and an improved alveolar disruption in PTX-treated rats compared to those in the BLM-instilled 28 d rats (Figure 2 A). Our results demonstrated that PTX can reduce the severity of subsequent pulmonary fibrosis.

**Table 1:** Lung-to-body weight ratio, the pulmonary inflammation and fibrosis scores.

Time	Treatment	Body weight (g)	Lung-to-body weight ratio	Pulmonary inflammation (Score)	Pulmonary fibrosis (Score)
od	Saline	220.90±6.39	0.68±0.04	0.00±0.00	0.00±0.00
7 d	Saline	246.75±8.15	0.79±0.01	0.00±0.00	0.00±0.00
	BLM	184.79±12.14*	1.53±0.10*	1.92±0.67*	1.33±0.49*
14 d	Saline	265.33±8.56	0.73±0.02	0.00±0.00	0.00±0.00
	BLM	204.36±13.04*	1.43±0.14*	2.25±0.45*	1.67±0.49*
	PTX	203.51±15.69	1.43±0.14	2.33±0.49	1.58±0.51
28 d	Saline	286.11±8.12	0.70±0.02	0.00±0.00	0.00±0.00
	BLM	222.75±13.35*	1.18±0.10*	2.5±0.52*	2.25±0.45*
	PTX	246.24±14.27**	1.09±0.08**	2.00±0.60**	1.83±0.58**

* *P*<0.05 versus saline-treated control,

** *P*<0.05 versus BLM-treated 28 d rats, n = 12.

Because collagen accumulation, an important indicator of lung fibrosis, can be reflected by the type-I collagen content and the hydroxyproline levels [29], we detected the changes of type-I collagen content and the hydroxyproline levels in rat lungs after BLM-instillation with/without PTX treatment. Our results revealed that rat lungs exposed to BLM showed a notable increase in type-I collagen deposition and expressed significantly high hydroxyproline levels as seven folds over untreated or saline-treated lungs (Figure 2 B, C), indicating that BLM increases collagen accumulation in fibrotic lung tissues. Interestingly, the collagen accumulation induced by BLM was suppressed about 40% in PTX-treated fibrotic lung tissues (Figure 2 B, C).

### Effect of PTX on suppressing TGF-β1/Smad3 pathway

**Figure 3:**
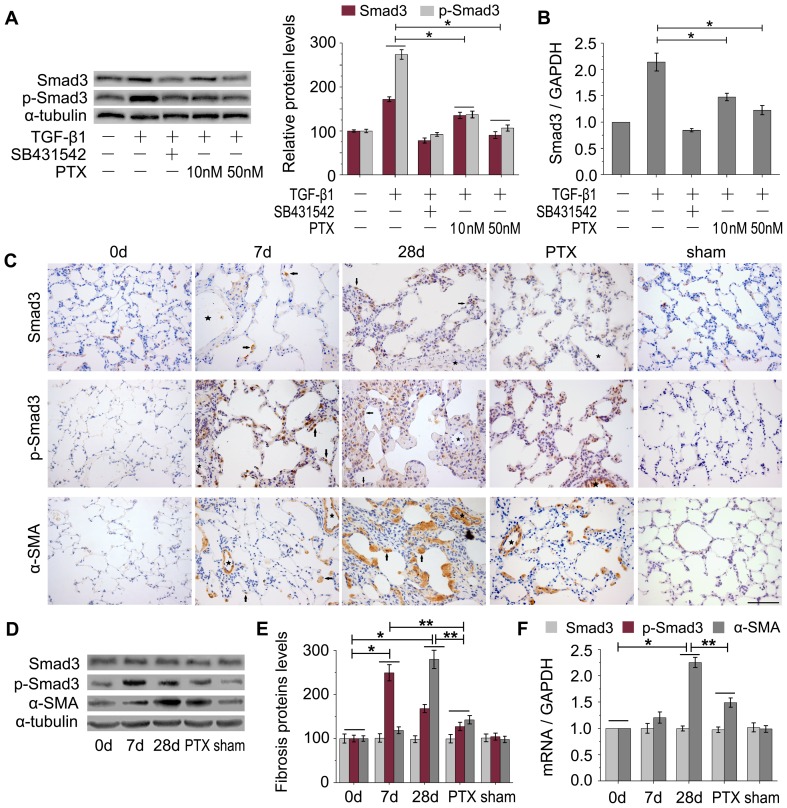
PTX suppresses the TGF-β1/Smad3 signaling pathway. A: Smad3 and p-Smad3 expression in A549 cells by western blot analysis. α-tubulin was used as loading controls. B: Quantitative RT-PCR analysis of Smad3 expression in A549 cells. GAPDH was used a control gene. In Fig. 3A,B, TGF-β1-treatment increased the expression of p-Smad3 and Smad3 in A549 cells, which could be reduced by PTX treatment. *P<0.05 versus TGF-β1 treatment group, n = 3 replicates. C: Immunohistochemical staining of Smad3, p-Smad3 and α-SMA in rat lung tissues. Black arrows, Smad3, p-Smad3 or α-SMA positive staining AECs. Stars, vasculature. Scale bars: 150 µm. A majority of Smad3, p-Smad3 and α-SMA staining was found to be localized in the AECs of fibrotic lungs. The BLM-untreated (0 d) or sham control lung tissues showed almost a total absence of α-SMA and p-Smad3. TGF-β1-treatment increased the expression of p-Smad3 and α-SMA in fibrotic lungs (7 d, 28 d), which was restored by PTX treatment to some extent. D: Western blot analysis. E: The ratios of Smad3 (p-Smad3, or α-SMA)/α-tubulin of Fig. 3D. α-tubulin was used as the internal standard. n = 3 replicates. F: Quantitative RT-PCR analysis of Smad3 and α-SMA expressions in rat lung tissues (n = 3 replicates). GAPDH was used as a control gene. In Fig. 3D–F: the quantity of p-Smad3 in lung tissues reached peaked on day 7 and the quantity of α-SMA in lung tissues reached peaked on day 28 after BLM treatment. PTX could reduce the expression of p-Smad3 and α-SMA obviously, but had no effect on Smad3. *P<0.05 versus 0 d. **P<0.05 versus PTX.

Smad3 and p-Smad3 are involved in TGF-β1-induced fibrotic gene responses as well as in BLM-induced pulmonary fibrosis, p-Smad3 is responsible for TGF-β1 fibrosis activation and alpha-smooth muscle actin (α-SMA) is one of the key indicators of fibrotic lung diseases [30]–[33]. Therefore, the effect of PTX on TGF-β1/Smad3 pathway was studied to explore the mechanism of PTX in relieving of pulmonary fibrosis. We found that TGF-β1 treatment resulted in an increase of Smad3 and p-Smad3 in A549 cells, and the p-Smad3 level was increased much higher than Smad3. However, the expression of Smad3 and p-Smad3 was remarkably reduced with PTX treatment (Figure 3 A, B), especially in 50 nM PTX-treated cells.

After exposure to BLM for 28 days, notable pulmonary fibrosis was observed, and strong staining of Smad3, p-Smad3 and α-SMA in AECs was also revealed as a prominent feature of BLM-induced rat lung tissues compared to BLM-untreated or sham control tissues (Figure 3 C). Western blot and quantitative real-time PCR showed a notable upregulation of p-Smad3 in the BLM-treated lungs, the p-Smad3 levels reached peaked on 7 day and α-SMA levels reached peaked on 28 day with BLM treatment (Figure 3 D–F). PTX could reduce the expression of p-Smad3 significantly, but had no obvious effect on Smad3 expression (Figure 3 D–F). The above results support PTX, in pulmonary fibrosis, could suppress the Smad3 and p-Smad3 activities obviously.

### Effect of PTX on upregulating miR-140

MiR-140 can suppress the TGF-β1 pathway through targeting Smad3 in the C3H10T1/2 and 3T3 cell lines [4], [5]. Our results showed that the TGF-β1 activity could be suppressed by PTX, which promotes us to examine the effect of PTX on miR-140 expression. In A549 cells, the miR-140 expression was significantly reduced after TGF-β1 treatment compared with untreated controls, but it was obviously increased after additional interference with TGF-β1 signal pathway blocker SB431542 (*P<0.05, Figure 1 D, E), suggesting that miR-140 correlated inversely with TGF-β1 activities. Moreover, PTX treatment dramatically increased the miR-140 expression compared to TGF-β1-treated cells, and slightly lower than that in untreated cells (**P<0.05, Figure 1 D, E).

In the lung tissues of BLM-treated rats, the miR-140 levels reached their nadir on day 7, approximately two-fold lower than that of in the untreated controls. A gradually increase of miR-140 level was observed with BLM-exposed time extension, between 21 d and 28 d with no more substantial changes, but still much lower than that in untreated or sham controls (*P<0.05, Figure 2 D, the 21 d data not shown). A pattern indicative of interstitial pneumonia on high-resolution CT and/or on histopathologic analysis of lung tissue obtained by lung biopsy is crucial for the final diagnosis [34]–[37]. Compared with healthy control lung tissues, much lower levels of miR-140 were further observed in pulmonary fibrotic tissues of patients (Figure 2E), who were diagnosed with pulmonary fibrosis using a high-resolution CT (HRCT) (Figure S3A) and on histopathologic analysis (Figure S3B). These results suggested that the downregulation of miR-140 might be an important event in fibrotic responses.

However, the miR-140 expression was restored to higher level with PTX treatment compared to only BLM-treated rats (**P<0.05, Figure 2 D). The above results demonstrate the miR-140 expression is negatively related to TGF-β1 activities, and miR-140 levels in the TGF-β1-stimulated A549 cells and BLM-induced rat lungs were dramatically upregulated by PTX treatment (**P<0.05, Figure 1 D, E and 2 D).

### MiR-140 suppressing TGF-β1/Smad3 pathway

**Figure 4:**
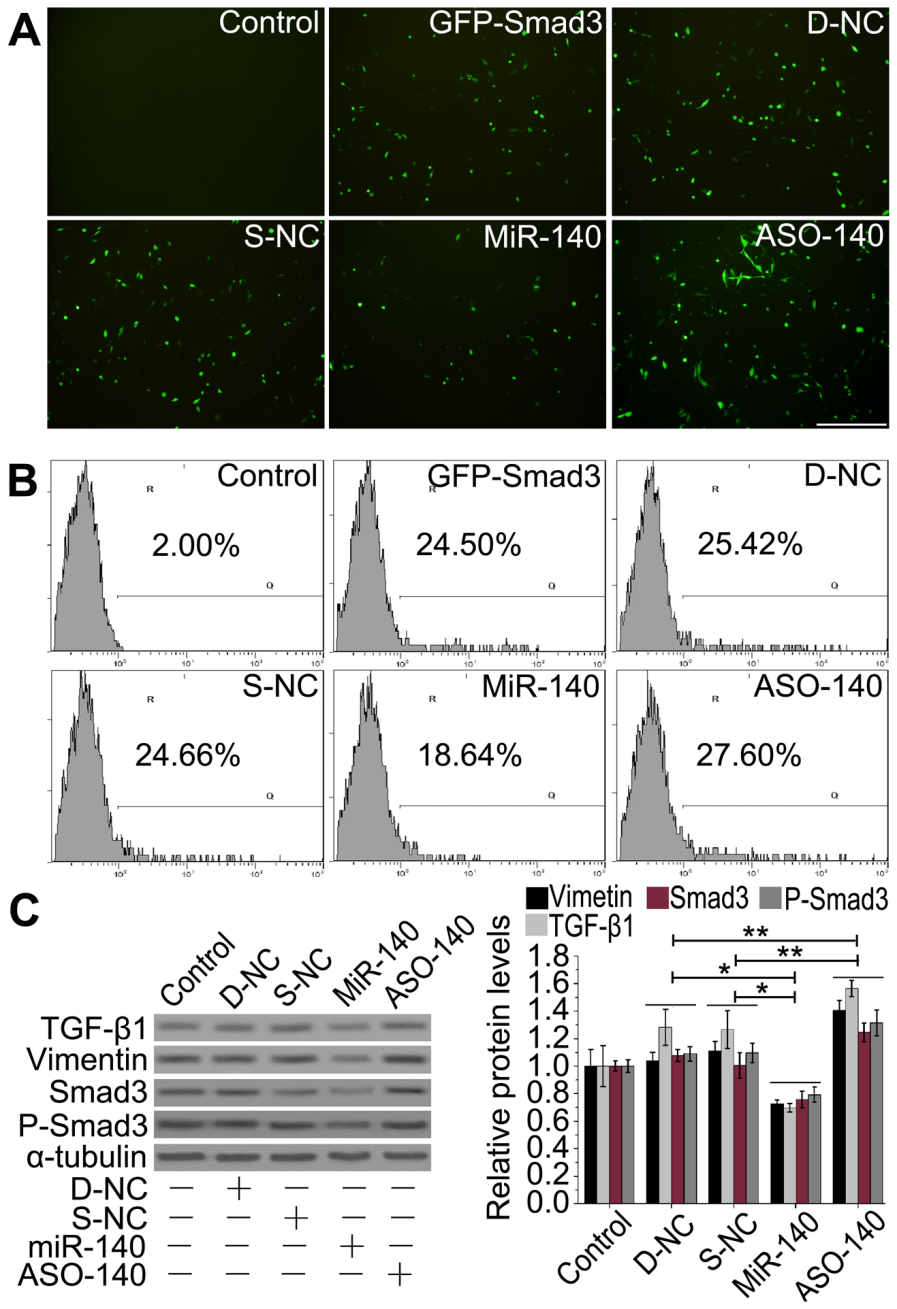
MiR-140 suppresses the TGF-β1/Smad3 pathway in A549 cells. A: Fluorescence microscope detection. Scale bars: 150 µm. B: Flow cytometry analysis. n = 3 replicates. Control, cells untreated with miRNA or plasmid; GFP-Smad3, cells treated with pcDNA-GFP-UTR; D-NC, cells treated with both D-NC (miR-140 mutation control) and pcDNA-GFP-UTR plasmid; S-NC, cells treated with both S-NC (the single-stranded RNA control) and pcDNA-GFP-UTR; miR-140, cells treated with both miRNA-140 and pcDNA-GFP-UTR; ASO-140, cells treated with both ASO-140 and pcDNA-GFP-UTR. In Fig. 4 A, B, GFP expression and the number of GFP-positive cells in miR-140-treated cultures were much lower than those in D-NC- or ASO-140-treated controls. C: Western blot analysis. The gene expression/α-tubulin ratios were shown on the right of gel. The miR-140 treatment led to an obvious reduction in the expression of TGF-β1, vimentin, Smad3 and p-Smad3 compared to the D-NC control groups. α-tubulin was used as loading controls. *P<0.05 versus miR-140 treatment, **P<0.05 versus ASO-140 treatment, n = 3 replicates.

Smad3 has been found to be a target for miR-140 in a pluripotent mouse embryonic fibroblast cell line [4], [5]. Smad3 also carries out fibrosis responses in epithelial cells [38], [39]. Then, we asked whether upregulation of miR-140 could suppress TGF-β1/Smad3 activities to further affect EMT in AECs. GFP-Smad3 plasmid (containing Smad3 mRNA 3′-UTR targeted by miR-140) was constructed to transfect A549 cells with miR-140. We found that a approximately 23% lower proportion of GFP-positive cells than that in the negative control (NC) groups, while it was a approximately 8.6% higher in antisense oligonucleotides (ASO)-140-treated cultures (Figure 4 A, B). These results confirm that miR-140 downregulates Smad3 expression by binding to Smad3-3′ UTR in AECs.

To further verify the roles of miR-140 and PTX in TGF-β1/Smad3 pathway, we studied whether overexpression of miR-140 would have similar effects as PTX on fibrosis-related protein expression. Our results showed that miR-140 overexpression led to a notable reduction in the expressions of fibrosis-related proteins (TGF-β1, vimentin, Smad3 and p-Smad3) compared to the NC controls (*P<0.05, Figure 4C), which was supported by other studies [31], [40], while these proteins levels were increased concomitant with a stellate and elongated fibroblast-like morphology after ASO-140 treatment (**P<0.05, Figure 4A, C). These results induced by miR-140 are similar to those affected by PTX treatment, which indicates miR-140 participates in the antifibrotic process by downregulating the expression of Smad3 and prevents its subsequent phosphorylation in AECs.

### PTX suppressing TGF-β1 pathway by upregulation of miR-140

**Figure 5:**
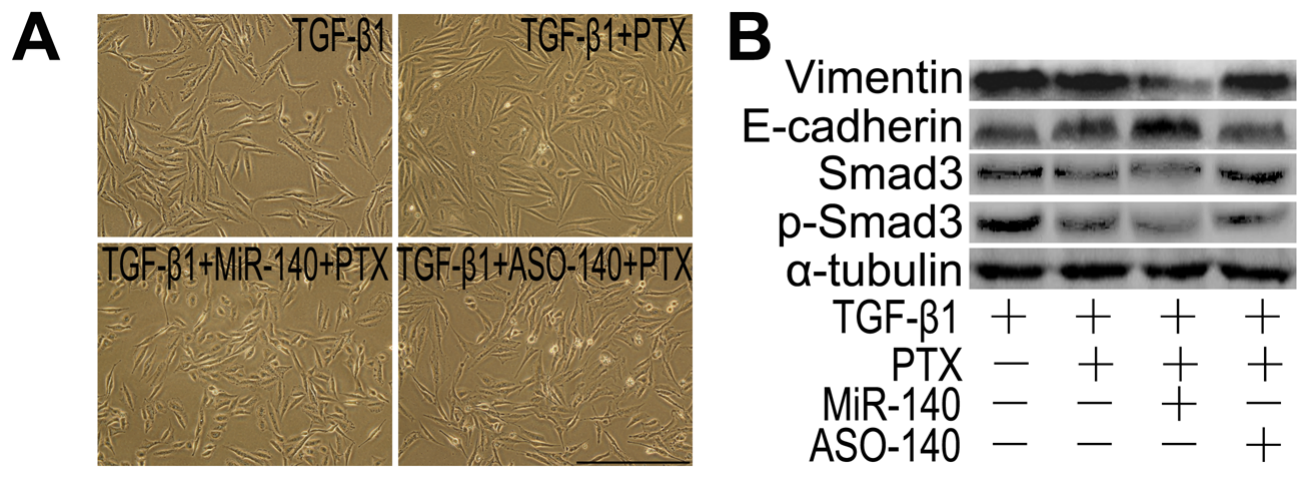
MiR-140 increased the sensitivity of AECs to PTX. A: Morphological characteristics. Scale bars: 150 µm. TGF-β1 was added into miR-140- or ASO-140-transfected A549 cells for 24 h prior to PTX (50 nM) treatment. A549 cells treated with TGF-β1 or ASO-140 presented a fibroblast-like morphology, and the fibroblast-like morphology was reversed to epithelial-like characteristics in PTX- or PTX+miR-140-treated cultures. B: Western blot analysis. The levels of vimentin, Smad3 and p-Smad3 levels were increased and E-cadherin was downregulated in TGF-β1-treated A549 cells, which were reversed in PTX-, or PTX+miR-140-treated cells, especially in both PTX and miR-140-treated cultures. n = 3 replicates.

To further confirm the effect of PTX on suppressing TGF-β1/Smad3 activities through miR-140, we investigated whether miR-140 overexpression would amplify the actions of PTX. TGF-β1-treated A549 cells underwent an EMT, leading to a fibroblast-like morphology with increased vimentin and Smad3/p-Smad3 levels, but downregulated E-cadherin level, which can be reversed by PTX (Figure 5 A, B). Furthermore, their morphologies and these protein levels became more close to those in both miR-140 and PTX treatment cells (Figure 5 A, B). Western blot further confirmed the expression changes of vimentin, Smad3/p-Smad3 and E-cadherin levels (Figure 5 B). However, the morphology kept fibroblast-like and the protein levels stayed abnormal in both ASO-140 and PTX treated cells.

**Figure 6:**
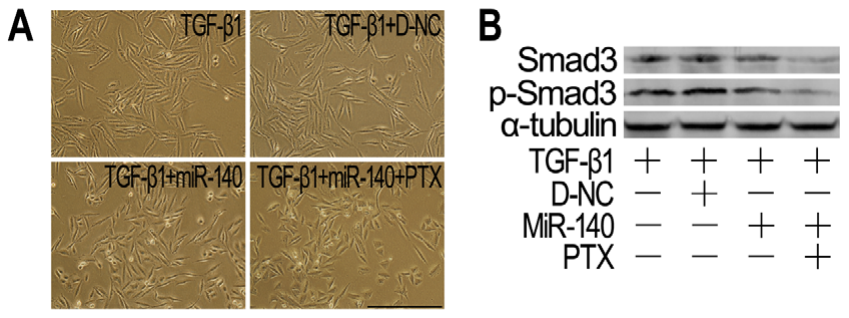
MiR-140 affected EMT and Smad3/p-Smad3 expression. A: Morphological characteristics. Scale bars: 150 µm. A549 cells treated with TGF-β1 or TGF-β1+ D-NC underwent a fibroblast-like morphology, while their morphologies were reversed by miR-140 or miR-140 plus PTX treatment. B: Western blot analysis. α-tubulin was used as loading controls. The Smad3 and p-Smad3 levels were decreased obviously in miR-140- or PTX+miR-140-treated A549 cells, especially in PTX+miR-140-treated cells, compared to those in TGF-β1- or TGF-β1+D-NC-treated cultures. n = 3 replicates.

**Figure 7:**
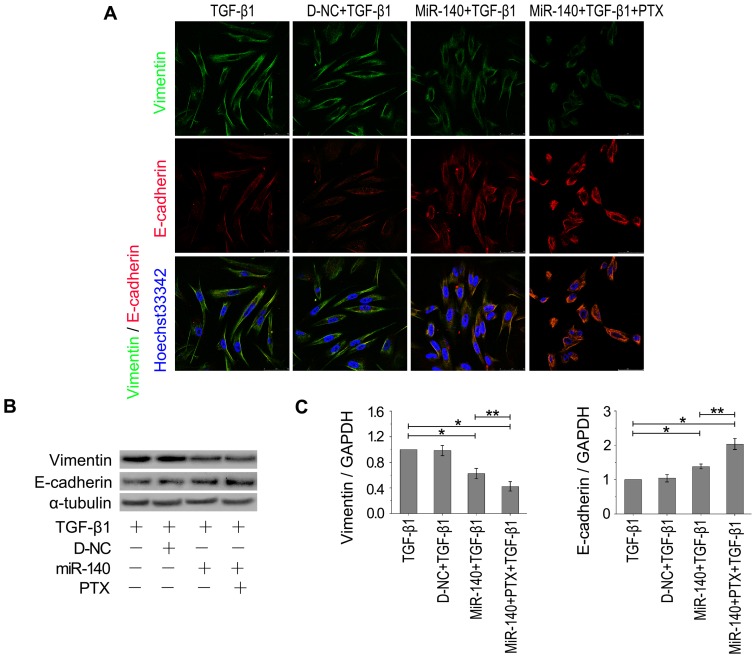
Regulation of miR-140 to EMT-related gene expression. A: Fluorescence images. Red, CY3-labeled E-cadherin. Green, FITC-labeled vimentin. Scale bars: 50 µm. B: Western blot analysis. C: Quantitative RT-PCR analysis. n = 3 replicates. E-cadherin expression was increased and vimentin was downregulated obviously in miR-140- or PTX+miR-140-treated cells, especially in PTX+miR-140-treated cultures, compared to TGF-β1- or TGF-β1+D-NC-treated controls. *P<0.05 versus TGF-β1-treated cells, **P<0.05 versus miR-140-treated cells, n = 3 replicates.

EMT-derived cells play important roles in promoting fibrogenesis [38]. Therefore, we investigated the effects of miR-140 and PTX on the EMT. TGF-β1 treatment resulted in a stellate and elongated fibroblast-like morphology, as well as Smad3 and p-Smad3 upregulation (Figure 6 A, B). Cells treated with both TGF-β1 and miR-140, in part, appeared elongated morphology to cobblestone-like morphology transition, and the levels of Smad3 and p-Smad3 were downregulated in these cells (Figure 6 A, B). Treatment with both miR-140 and PTX would lead to a cobblestone-like epithelial morphology and the lowest levels of Smad3/p-Smad3 in TGF-β1-treated cells (Figure 6 A, B). Moreover, both miR-140 and PTX treatment resulted in a better reversal of the TGF-β1-induced E-cadherin and vimentin expression (Figure 7 A–C). Together, these results support that miR-140 increases the efficiency and sensitivity of the reversal effect of PTX on EMT.

## Discussion

Previous studies reported that PTX could ameliorate hepatic and renal fibrosis [7]–[10]. In this study, we investigated the mechanism of PTX in improving pulmonary fibrosis. Our results demonstrated that low-dose PTX reversed the TGF-β1-induced EMT in A549 cells as well as RLE-6TN cells, and improved the BLM-instilled pulmonary fibrosis in rat lungs with miR-140 upregulation and Smad3/p-Smad3 diminishment. This study provides the evidence of an important role of low-dose PTX in ameliorating EMT and pulmonary fibrosis by upregulating miR-140 to suppress the activities of TGF-β1/Smad3 pathway.

A growing body of evidence indicates that lung fibrotic pathogenesis is driven by abnormally activated AECs [41]–[43]. AECs secrete and TGF-β1 activation would promote EMT and the differentiation of fibroblasts into myofibroblasts [41], [42], and serve as chemoattractants for inflammatory cells and fibroblasts to contribute to intra-alveolar fibrosis [43]. Kim et al. revealed AECs are the main source of mesenchymal expansion [44]. Epithelial injury, apoptosis, and an increased number of hyperplastic and hypertrophic type II pneumocytes characterize lungs affected by pulmonary fibrosis. Moreover, AEC II is viewed as progenitor for fibroblasts in vivo [42], [45]. A549 cells are one of the human AEC II-derived cell lines, which retain the features and metabolic properties of type II cells [46]. Our results showed that the TGF-β1-treated A549 cells exhibited downexpression of E-cadherin and overexpression of vimentin, suggesting that dys-regulation of AEC II contributes to the characteristic aberrant repair process and the pathogenesis of pulmonary fibrosis.

PTX, a stabilizer of cellular MTs, inhibits the collagen-induced arthritis and the fibrosis-related systemic sclerosis, and ameliorates hepatic fibrosis, tubulointerstitial fibrosis as well as renal fibrosis [6]–[10]. Many studies reported that pulmonary fibrosis-derived fibroblasts or myofibroblasts synthesized significantly more extracellular matrix components like type-I collagen [47]–[50] than did normal lung tissue-derived fibroblasts. Hydroxyproline levels also reflect the extent of collagen deposition in fibrotic tissues [29], [51]. Therefore, we detected the effect of PTX on changing of hydroxyproline levels and the type-I collagen deposition in rat lungs. Our results demonstrated that type-I collagen deposition and the hydroxyproline levels were increased significantly in BLM-treated lungs than those in saline-treated (sham) or BLM-untreated control lungs. The enhanced type-I collagen deposition and hydroxyproline levels in BLM-treated lungs can be obviously ameliorated by PTX treatment, suggesting an antifibrotic effect of PTX.

EMT, converting from epithelial phenotype to fibroblastic phenotype, plays an prominent role in both experimental and clinical pulmonary fibrosis [44], [45], [52]. Emerging evidence shows that the colocalization of both epithelial and mesenchymal marker cells undergo EMT in lung tissue from IPF patients [44], [45], [53], [54]. EMT also accounted for about 33% of fibroblasts in experimental lung fibrosis in mice [55]. Furthermore, the significance of EMT in the pulmonary fibrogenic process may include direct generation of fibroblasts and indirect effects on releasing of cytokines by injured epithelial cells, which creates a profibrogenic environment [38], [44]. During EMT, the diminished E-cadherin level and upregulation of TGF-β1, vimentin, Smad3 and p-Smad3 levels are concomitant with the ability of epithelial cells to adopt mesenchymal phenotypes [31], [32], [56]. As expected, we found that PTX treatment resulted in EMT phenotypic reversion and normalization of TGF-β1, vimentin, E-cadherin, Smad3 and p-Smad3 expression. A549 cells treated with PTX, in majority, are resistant to the TGF-β1-induced EMT, whereas PTX-untreated controls are more vulnerable to EMT. Because early treatment was often missed in pulmonary fibrosis clinical therapy, we used the mid-term administration (PTX treatment started at 14 d) to mimic the majority of the clinical medical record. Low-dose PTX (0.6 mg/kg) was found to significantly attenuate the histological and biochemical signs of BLM-induced pulmonary fibrosis, including epithelial degeneration, collagen deposition and several fibrogenic cytokines, such as TGF-β1, α-SMA, Smad3 and p-Smad3, supporting that PTX ameliorated pulmonary fibrosis by suppressing TGF-β1 pathway.

TGF-β1 is responsible to trigger a cascade of events leading to fibrosis, and the signaling activity of Smad3 is modulated through phosphorylation as well as cytosol-nucleus translocation [32], [57]. Because EMT induced by TGF-β1 is a key issue in the pathogenesis of tissue or organ fibrosis [11]–[13] and inhibition of TGF-ß1 or Smad 2/3 could reverse EMT in hepatic fibrogenesis [58], drugs or targets of TGF-β/Smad3 pathway might be a suitable approach for pulmonary fibrosis therapy. Our results showed that PTX treatment not only suppressed TGF-β1-activated Smad3/p-Smad3 at mRNA or protein levels to further reverse subsequent EMT in A549 cells, but also downregulated p-Smad3 levels to improve subsequent pulmonary fibrosis in rat lungs. Our finding was supported by previous study, which demonstrated low-dose PTX blocked TGF-β1-induced myogenesis in C2C12 myoblasts [59]. Low-dose PTX can also interrupt fibrogenic TGF-β1 signaling between gallbladder epithelial cells and myofibroblasts [60]. Similarly, we found that low-dose PTX (10–50 nM in vitro and 0.6 mg/kg in vivo) resulted in a normalization of TGF-β1/Smad3 aberrance. These results support that PTX ameliorates EMT and pulmonary fibrosis by suppressing TGF-β1/Smad3 and p-Smad3 activities.

MiR-140 expression is downregulated in low grade liver fibrosis [61]. MiR-140 can negatively regulate the expression of Smad3 [4], [5], which acts as a key factor in TGF-β1-mediated EMT [32] and the fibrosis response [30]–[32], [57]. These reports imply that downregulation of miR-140 would promote pulmonary fibrosis. Similarly, we found that there was a notable reduced expression of miR-140 during TGF-β1-treated EMT in A549 cells, BLM-induced pulmonary fibrosis in rat lungs and lung samples of IPF patients. Moreover, PTX could block the action of TGF-β1 by upregulating miR-140. If miR-140 is a major leader to prevent pulmonary fibrosis, it may either precede or coincide with the synthesis of TGF-β1 [62]. Our results revealed that miR-140 is downregulated in BLM-induced pulmonary fibrosis, which is negatively related with TGF-β1 activities. We also found that overexpression of miR-140, same as PTX treatment, could reverse TGF-β1-induced EMT in A549 cells by increasing the E-cadherin level and decreasing the expression of vimentin, Smad3 and p-Smad3. Moreover, our results reveal that miR-140 overexpression increases the sensitivity of AECs to PTX, but more precise mechanisms of cooperation of PTX and miR-140 in suppressing TGF-β1/Smad3-induced pulmonary fibrosis need further study.

In summary, our results demonstrate that low-dose PTX prevents EMT transition and ameliorates pulmonary fibrosis by upregulating miR-140 expression to further suppress the TGF-β1/Smad3 pathway, which highlighted a new way for pulmonary fibrosis therapy.

## Materials and Methods

### Ethics statement

All animal experimental procedures in this study were performed in accordance with the Institutional Animal Care and followed the National Institutes of Health Guide for the Care and Use of Laboratory Animals (Maryland, USA). The protocol was approved by the Committee on the Ethics of Animal Experiments of Binzhou Medical University (Permit Number: SCXK 2009 0009).

The patients' study was approved by the Ethics Committee of the Affiliated Hospital of Binzhou Medical University. Written informed consent was obtained from all the participants.

### Cell culture

Human type II alveolar epithelial cells (A549) were purchased from the Cell Bank of Chinese Academy of Sciences. RLE-6TN cells were purchased from XiangYa Central Experiment Laboratory. The cells were maintained in cell culture medium as described [56]. Confluent cultures of cells were maintained in RPMI-1640 (Hyclone, USA) containing 10% FBS, 100 U/ml penicillin and 100 µg/ml streptomycin at 37°C in a humidified 5% CO_2_ atmosphere. Confluent cultures of cells were maintained in serum-free RPMI-1640 containing 0.1% FBS for 24 h prior to stimulation with cytokines. In PTX-treated experiments, A549 cells were pre-incubated with exogenous TGF-β1 for 24 h before treatment with PTX, then were co-incubated for 48 h. As the positive control, a specific inhibitor of the TGF-β1 receptor type I kinase (ALK5)-SB431542 hydrate (5 µmol/L) was used to pretreat cells for 30 min before treatment with TGF-β1 and co-incubated for 72 h. In miRNA experiments, the miRNA-transfected cells were stimulated with TGF-β1 (5 ng/ml) for 24 h before PTX treatment and then co-cultured in serum-free RPMI-1640 containing 0.1% FBS for 48 h. The cells were then harvested and lyzed. All experiments were performed three replicates.

### Immunofluorescence analysis and western blot analysis

Immunofluorescence analysis and western blot analysis were performed according to a standard method, as described previously [63]. Fluorescence images were captured under a laser scanning confocal microscope (LSCM, Leica Company, Germany) at room temperature. To ensure equal loading in western blot, the proteins were normalized to α-tubulin (Sigma-Aldrich, St. Louis, MO, USA).

### Type-I collagen ELISA

The type-I collagen levels in rat lung tissues were determined using a rat collagen type I (Col I) ELISA kit (R&D Systems, Abingdon, UK) and performed according to the manufacturer's instructions. Briefly, Purified Rat collagen I antibody was used to coat microtiter plate wells. Then the tissue lysis was added to wells. Second antibody with HRP labeled was combined and TMB was added. Reaction was determinated using a wavelength of 450 nm. The concentration of collagen I in the samples was then analyzed by comparing the O.D. of the samples to the standard curve.

### Hydroxyproline assay

A hydroxyproline assay was performed to assess collagen synthesis as follows: Rats were anesthetized, and the left lung was excised. Lung tissues were dried at 110°C for 24 hours. 6**′-**N hydrochloric acid in the absence of oxygen was used to acid hydrolyzation. Vials were vacuum-sealed and incubated at 110°C for 24 hours. Hydroxyproline content was quantified as described previously [64], [65] and expressed as µg/mg lung weight.

### Animals and BLM administration

Adult male SD rats weighing 220 g±10 g were purchased from the Yantai Green Leaf Experimental Animal Center. The animals were housed at 22°C–25°C at 65%–70% humidity on a 12 h light-dark cycle and had free access to food and water. All experimental procedures were performed in accordance with the Institutional Animal Care and followed the National Institutes of Health Guide for the Care and Use of Laboratory Animals (Maryland, USA).

Rats were randomly divided into five groups: 0 d group, the rats were untreated; 7 d group, the rats were given a single intratracheal instillation of 50 mL saline containing BLM (5 mg/kg; Sigma-Aldrich, St. Louis, MO, USA) or a single 50 mL saline alone one time, and were killed at the 7^th^ day post-treatment; 28 d group, the rats were given a single intratracheal instillation of 50 mL saline containing BLM (5 mg/kg), and were killed at the 28^th^ day post-treatment; PTX group, the BLM-instilled rats treated with low-dose PTX (0.6 mg/kg; Sigma-Aldrich, St. Louis, MO, USA) starting 15^th^ day after the intratracheal injection of BLM, intravenously (tail vein) daily for 14 days; sham group, the rats were given a single intratracheal instillation of 50 mL saline alone, and were killed at the 28^th^ day post-treatment; saline+PTX group, the saline-instilled rats treated with 0.6 mg/kg PTX starting 15^th^ day after the intratracheal injection of saline, intravenously (tail vein) daily for 14 days. The severity of lung fibrosis and the effect of PTX on pulmonary fibrosis were assessed using a histological evaluation, collagen delineation and the pathological score as previous report [66].

### Morphological studies

Paraformaldehyde-fixed lung tissues were embedded with paraffin and stained with haematoxylin and eosin (HE) for histopathologic examination. Masson's trichrome and immunohistochemistry were performed to detect α-SMA, Smad3 and p-Smad3. These methods were performed as previously described [67]. We evaluated the effect of PTX on pulmonary fibrosis by histological evaluation using the Szapiel's score of pulmonary fibrosis [66]: 0, no fibrosis; 1, <20% of alveolar area affected; 2, 20–50% affected; 3, >50 affected. At least 20 randomly selected microscopically fields, from sections of the distal lung of each rat, were examined by three blinded investigators.

### Quantitative real-time polymerase chain reaction (qRT-PCR)

Total RNA was extracted, purified and retro-transcribed as described previously [68]. Quantitative Real-time PCR was performed on a BioRad real-time PCR instrument (BioRad, USA) using an iQ SYBR Green Supermix with Opticon (MJ Research Inc., Waltham, MA, USA). U6/GAPDH was used as the positive control. The specific primers were shown as follows: Has/Rno-mir-140-FO, 5′-GATGCTCACAGTGGTTTTACCC-3′; Has/Rno-miR-140-RE, 5′-TATCGT-TGTTCTGCTCTCTGTCTC-3′; and U6-FO: 5′-ATTGGAACGATACAGAGAAGATT-3′; U6-RE: 5′-GGAACGCTTCACGAATTTG-3′. The melting curve data were collected to check PCR specificity. MiR-140 expression was normalized against U6 and other regulated genes were normalized with GAPDH. Primers and probes were provided from GenePharma. Expression level was calculated by ΔΔCt method, and fold changes were obtained using the formula 2^−ΔΔCt^. The primers used to detect Smad3 as follows: 5′-AGCACACAATAACTTGGACC-3′ (forward), 5′-TAAGACACACTGGAACAGCGGATG-3′ (reverse). α-SMA primers: 5′-ACTGGGACGACATGGAAAAG-3′ (forward), 5′-CATCTCCAGAGTCCAGCACA-3′ (reverse). GAPDH: 5′-TGCTGAGTATGTCGTGGAGTCTA-3′ (forward), 5′-AGTGGGAGTTGCTGTTGAAATC-3′ (reverse). All experiments were performed three replicates.

### Construction of reporter plasmid UTR and miRNA transfection

Green fluorescent protein (GFP) was digested from pEGFP-N1 (Clontech, USA) with BamHI/NotI (blunted) and then cloned into pcDNA3.1/Zeo (+) (Invitrogen, USA), and the digested with BamHI (blunted) to form the pcDNA-GFP vector. The 3′-UTRs (2550 bp) of Smad3 were cloned via polymerase chain reaction (PCR) in an Eppendorf cycler using a program consisting of 28 cycles of denaturation at 94°C for 45 s, annealing at 52°C for 45 s, and elongation at 72°C for 120 s. The 3′-UTRs were cloned into the T vector (Takara, Japan) to construct the T-UTR vector. To obtain the pcDNA-GFP-UTR vector, the 3′-UTRs of Smad3 were cut from the T-UTR vector and then cloned to the downstream of GFP in the pcDNA-GFP plasmid using EcorI/XhoI, which was named as GFP-Smad3. The primers were 5′-TGGAACTCTACTCAACCCATTG-3′ (forward) and 5′-TACATACGCCCAAAGCACCT-3′ (reverse). MiR-140, D-NC (miR-140 mutation control with mutation seed sequences), ASO-140 (specific to miR-140 sequence) and S-NC (the single-stranded RNA negative control) were cooperated with pcDNA-GFP-UTR vector, respectively, to transfect A549 cells using Lipofectamine 2000 transfection reagent (Invitrogen, USA) according to the manufacturer's instructions. The sequences of oligos were shown in Table 2. The samples were collected at 24 h after transfection. The GFP-positive cells and the EMT protein expression were confirmed via a fluorescence microscope, flow cytometry measurements (FCM; Beckman Coulter, Inc., USA) or western blot analysis.

**Table 2:** The sequences of chemically synthesized oligos.

Oligos		Sequence (5′→3′)
miR-140^a^	sense	CAGUGGUUUUACCCUAUGGUAG
	antisense	CUACCAUAGGGUAAAACCACUGUU
D-NC^a^	sense	C**GC**U**U**GUUUUACCCUAUGGUAG *
	antisense	CUACCAUAGGGUAAAACCAACGUU
ASO-140	antisense	CUACCAUAGGGUAAAACCACUGU
S-NC	antisense	CAGUACUUUUGUGUAGUACAA

a The selected miRNAs were chemically synthesized in the form of small interfering RNA (siRNA) duplexes.

* the bold and underlined letters were the mutation sites of miRNA.

### Patients

All patientss with pulmonary fibrosis, between 50 to 80 years of age at presentation, were from the Affiliated Hospital of Binzhou Medical University between 2012 and 2013. The indication to perform lung biopsies with fiber bronchoscope or thoracic surgery had been made by the clinicians on the basis of the available guidelines. All patients have given written consent to surgical procedures and scientific evaluation of data. HRCT scans were performed during suspended inspiration, with patients in the supine position. HRCT scans were evaluated by three chest experienced radiologists, the radiological pattern was categorized as a typical IPF pattern with subpleural ground-glass opacity, reticular pattern, honeycombing and architectural distortion. Surgical lung biopsies were reviewed by three pathologists who were blind to the clinical information. Histopathological evidence of IPF was determined according to the American Thoracic Society and European Respiratory Society Consensus Classification criteria.

### Statistical analysis

Data were expressed as means ± SD. A one-way analysis of variance (ANOVA) and a factorial design variance analysis were performed. The statistical differences between the groups were determined using Student-Newman-Kuels (SNK) Test. *P*<0.05 was considered statistically significant. All experiments were performed at least three replicates.

## Supporting Information

Figure S1 **PTX ameliorates EMT in RLE-6TN cells.** Cell morphological changes. Under control conditions, cells exhibit cobblestone appearance typical of epithelial morphology. While following treatment with TGF-β1, loss of cell-cell contacts and acquisition of fibroblast-like morphology are seen. PTX attenuates TGF-β1-induced changes and maintains epithelial morphology. Scale bars: 150 µm.
(TIF)

Figure S2 **The effect of PTX on lung phenotype.** A: only saline treatment. B: treatment with both saline and PTX. The lung phenotype and the number of Smad3-/p-Smad3-positive cells in the saline+PTX treated lung tissues were similar to those in only saline-treated lungs using HE, Masson's trichrome and immunohistochemical analysis. Scale bars: 150 µm.
(TIF)

Figure S3 **The effect of PTX on lung phenotype.** A: High-resolution CT (HRCT) detection. HRCT showed that the presence of patchy, subpleural ground-glass opacities, reticular pattern, honeycombing and architectural distortion in the fibrotic lungs. B: Histopathological HE staining. The hyperplasia of alveolar type II cells, thickened alveolar walls, alveolar disruption and excessive ECM deposition were found in pulmonary fibrotic lungs. a–e: healthy control lungs, f–j: human pulmonary fibrosis lungs. Scale bars: 300 µm.
(TIF)
