# Supplementary figures and images for: Low-Dose Paclitaxel Ameliorates Pulmonary Fibrosis by Suppressing TGF-β1/Smad3 Pathway via miR-140 Upregulation
Source: PLoS One. 2013 Aug 15;8(8):e70725. doi: 10.1371/journal.pone.0070725 (PMC3744547; doi:10.1371/journal.pone.0070725)

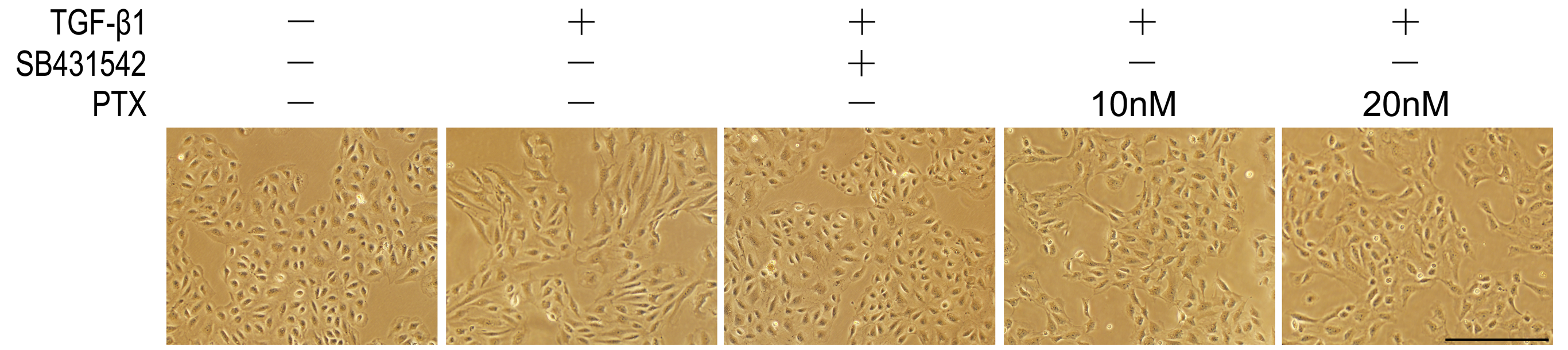

Supplement: Figure S1 — PTX ameliorates EMT in RLE-6TN cells. Cell morphological changes. Under control conditions, cells exhibit cobblestone appearance typical of epithelial morphology. While following treatment with TGF-β1, loss of cell-cell contacts and acquisition of fibroblast-like morphology are seen. PTX attenuates TGF-β1-induced changes and maintains epithelial morphology. Scale bars: 150 µm. (TIF) [file pone.0070725.s001.tif]

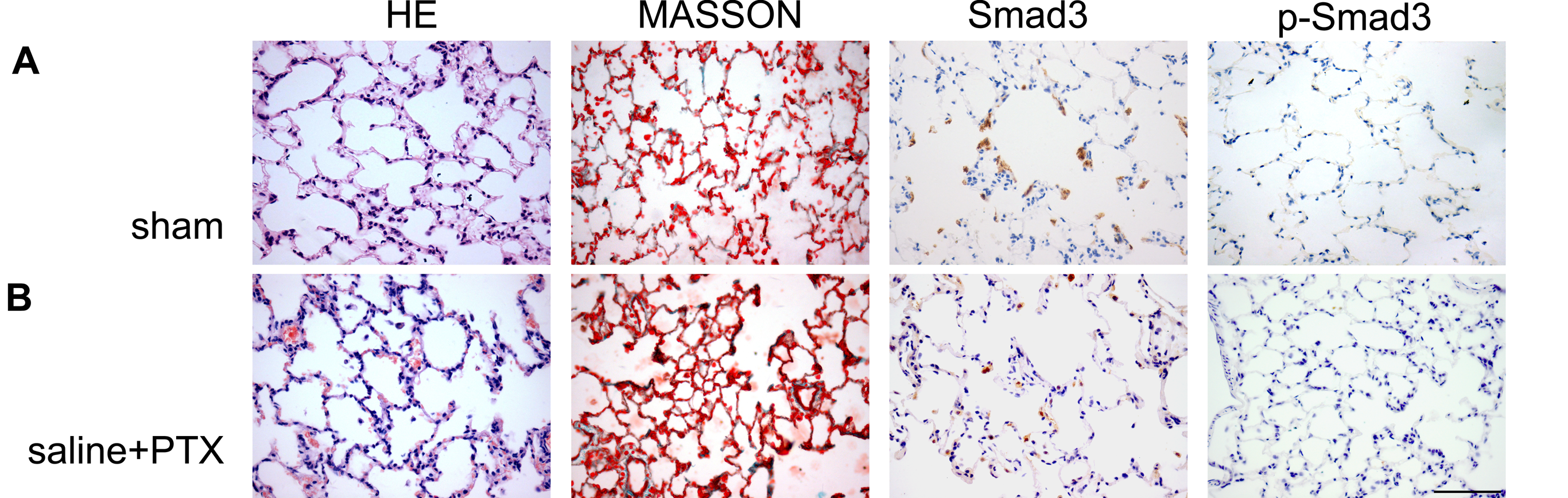

Supplement: Figure S2 — The effect of PTX on lung phenotype. A: only saline treatment. B: treatment with both saline and PTX. The lung phenotype and the number of Smad3-/p-Smad3-positive cells in the saline+PTX treated lung tissues were similar to those in only saline-treated lungs using HE, Masson's trichrome and immunohistochemical analysis. Scale bars: 150 µm. (TIF) [file pone.0070725.s002.tif]

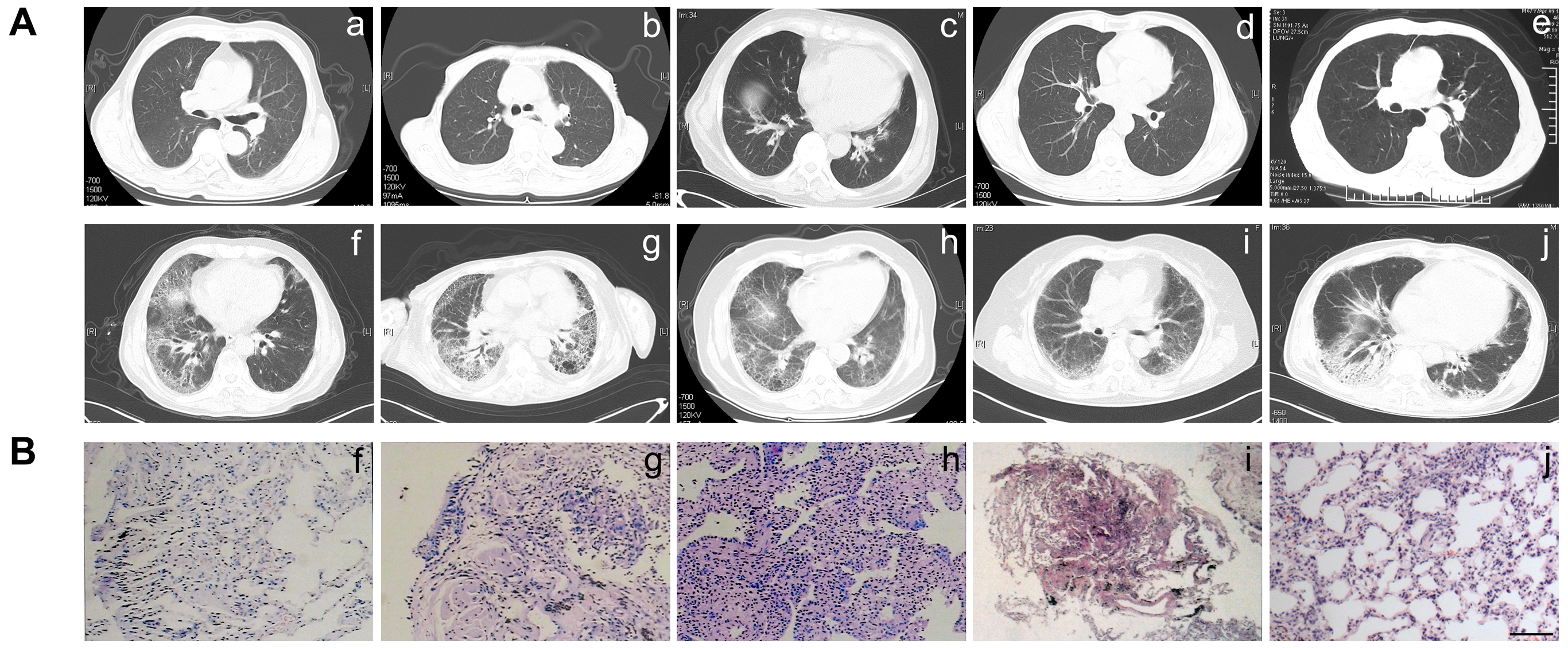

Supplement: Figure S3 — The effect of PTX on lung phenotype. A: High-resolution CT (HRCT) detection. HRCT showed that the presence of patchy, subpleural ground-glass opacities, reticular pattern, honeycombing and architectural distortion in the fibrotic lungs. B: Histopathological HE staining. The hyperplasia of alveolar type II cells, thickened alveolar walls, alveolar disruption and excessive ECM deposition were found in pulmonary fibrotic lungs. a–e: healthy control lungs, f–j: human pulmonary fibrosis lungs. Scale bars: 300 µm. (TIF) [file pone.0070725.s003.tif]
